# A note on mate allocation for dominance handling in genomic selection

**DOI:** 10.1186/1297-9686-42-33

**Published:** 2010-08-11

**Authors:** Miguel A Toro, Luis Varona

**Affiliations:** 1 ETS Ingenieros Agrónomos, 28040 Madrid, Spain; 2 Facultad de Veterinaria, Universidad de Zaragoza, 50013 Zaragoza, Spain

## Abstract

Estimation of non-additive genetic effects in animal breeding is important because it increases the accuracy of breeding value prediction and the value of mate allocation procedures. With the advent of genomic selection these ideas should be revisited. The objective of this study was to quantify the efficiency of including dominance effects and practising mating allocation under a whole-genome evaluation scenario. Four strategies of selection, carried out during five generations, were compared by simulation techniques. In the first scenario (MS), individuals were selected based on their own phenotypic information. In the second (GSA), they were selected based on the prediction generated by the Bayes A method of whole-genome evaluation under an additive model. In the third (GSD), the model was expanded to include dominance effects. These three scenarios used random mating to construct future generations, whereas in the fourth one (GSD + MA), matings were optimized by simulated annealing. The advantage of GSD over GSA ranges from 9 to 14% of the expected response and, in addition, using mate allocation (GSD + MA) provides an additional response ranging from 6% to 22%. However, mate selection can improve the expected genetic response over random mating only in the first generation of selection. Furthermore, the efficiency of genomic selection is eroded after a few generations of selection, thus, a continued collection of phenotypic data and re-evaluation will be required.

## Background

Estimation of non-additive genetic effects in animal breeding is important because ignoring these effects will produce less accurate estimates of breeding values and will have an effect on ranking breeding values. As a consequence, including these effects will produce a more accurate prediction and, therefore, more genetic response. This potential increase of genetic response is about 10% for traits with a low heritability, high proportion of dominance variance, low selection intensity and high percentage (>20%) of full-sibs [1].

However, dominance effects have rarely been included in genetic evaluations. The reasons, that can be argued, are the greater computational complexity and the inaccuracy in the estimation of variance components (it is commonly believed that 20 to 100 times more data are required including a high proportion of full-sibs [2]). It has also been claimed that there is little evidence of non-additive genetic variance in the literature (see for example [3]). However, although estimates are scarce, dominance variance usually amounts to about 10% of the phenotypic variance [4]. Furthermore, in an extensive review [5], estimates of the ratio of additive to dominance variance have been reported in wild species i.e. about 1.17 for life-history traits, 1.06 for physiological traits and 0.19 for morphological traits. In the same study, the estimate of this ratio for domestic species was 0.80.

Moreover, mating plans (or mating allocations) have been used in animal breeding for several reasons: a) to control inbreeding; b) in situations where economic merit is not linear; c) when there is an intermediate optimum (or restricted traits); d) to increase connection among herds and, finally, e) to profit from dominance genetic effects. With respect to the last point, it is well known that every methodology pretending to use non-additive effects [6-8] must contemplate two types of mating: a) matings from which the population will be propagated; b) matings to obtain commercial animals. Among all the methodologies aimed at profiting from dominance, mating allocation could be the easiest option. Optimal mating allocation relies on the idea that although selection should be carried out on estimated additive breeding values, animals used for commercial production should be the product of planned mating which maximizes the overall (additive plus dominance effects) genetic merit of the offspring. Mating allocation profits from dominance when the commercial population is constructed, but for the next generation only additive effects are transmitted.

Although not considered here, other ideas could be used to exploit dominance in later generations. The key idea is that selection should be applied not only to individuals and should be extended to mating. Although it is usually thought that application of the above ideas requires two separate lines as in the classical crossbreeding programmes or in the so-called reciprocal recurrent selection, it can be carried out in a single population [6,7]. Furthermore, a 'super-breed' model can be implemented to exploit both across- and within-breed dominance variances [9].

With the recent availability of very dense SNP panels and the advent of genomic selection [10] it seems natural that methods using dominance variation should be revisited. The aim of this study was to quantify the efficiency of mating allocation under a whole-genome evaluation scenario in terms of genetic response to selection in the first and subsequent generations.

## Methods

### Simulation data

A population was simulated for 1000 generations at an effective size of 100. After 1000 generations, the actual size of the population increased up to 1000 (500 per sex) and remained at 1000 for three discrete and consecutive generations. During the whole process, all individuals were generated with one gamete from a random father and one from a random mother. Therefore the data set for the estimation of the marker effects consisted of the 3000 individuals from the last three generations. These 3000 (generation 1001, 1002 and 1003) individuals were genotyped and phenotyped and then used as training population to estimate additive and dominance effects of SNP.

The genome was assumed to consist of 10 chromosomes each 100 cM long and 1000 loci/chromosome (i.e. a total of 9000 SNP plus 1000 QTL) were located at random map positions. Both SNP and QTL were biallelic. Mutations were generated at a rate of 2.5 × 10^-3 ^per locus per generation at the marker loci and at a rate of 2.5 × 10^-5 ^at the QTL loci. These mutation rates, taken from [10] are unrealistic but they seem to provide a reasonable level of segregation after only 1000 generations. Both the additive and the dominance effects were sampled from a standard normal distribution and scaled to obtain the desired values of h^2 ^(V_A_/V_P_) and d^2 ^(V_D_/V_P_) where V_A_, V_D _and V_P _the additive, dominance and phenotypic variances as defined in, for example [11]. The simulation of additive and dominance effects was a bit simplistic because it is known that the distribution of additive effects is leptokurtic and the distribution of dominance effects is dependent on additive effects [12]. In generation 1, about half of the loci were fixed for allele 1 and the other half were fixed for allele 2.

### Model of analysis

For simplicity, estimation of marker effects was carried out using a Bayes A method [10] with two alternative models:

a) The first model assumed that the phenotypic value of individual j (j = 1, ... N) is

$${\text{y}}_{\text{j}}=\mu +\sum_{i=1}^{p}x_{ij}{\text{a}}_{i}+{\text{e}}_{\text{j}}$$

where p is the number of SNP and x_ij _are indicator functions that take the values 1, 0, -1 for the SNP genotypes AA, Aa and aa at each loci, respectively. The assumed distributions for each additive a_i _component and residual component (e_j_) were:

$$\begin{array}{c} {\text{a}}_{i}\;\sim \;N(0,\sigma_{ai}^{2})\\ \text{and }e_{j}\sim \;N(0,\sigma_{e}^{2})\;. \end{array}$$

The prior distribution of the variances was the scaled inverted chi-square distribution:

$$\begin{array}{c} \;\sigma_{ai}^{2}\sim \chi^{-2}(v,S)\\ \text{and }\;\sigma_{e}^{2}\sim \chi^{-2}(-2,0) \end{array}$$

where *S *is a scale parameter and *v *is the number of degrees of freedom. The values of *v *= 4.012 and *S *= 0.0020 were taken from [10].

b) The second model also assumed, in addition, that dominance effects were included for each SNP:

$${\text{y}}_{\text{j}}=\mu +\sum_{i=1}^{p}x_{ij}{\text{a}}_{i}+\sum_{i=1}^{p}w_{ij}{\text{d}}_{i}+{\text{e}}_{\text{j}}$$

where w_ij _are indicator functions that take the values 0,1, 0 for the SNP genotypes AA, Aa and aa, respectively. The assumed distributions for each dominance effect (d_i_) was:

$${\text{d}}_{i}\;\sim \;N(0,\sigma_{di}^{2}).$$

The prior distribution of the variances of the dominance effects was the scaled inverted chi-square distribution

$$\sigma_{di}^{2}\sim \chi^{-2}(v,S)$$

where *S *is a scale parameter and *v *is the number of degrees of freedom. As before, the values of *v *= 4.012 and *S *= 0.0020 were assumed.

Gibbs sampling based on posterior distributions conditional on other effects was implemented for estimation by averaging the samples from 10,000 cycles, after discarding the first 1,000.

### Prediction of breeding values

From the estimates of additive and dominance effects, breeding values (u_i_) were calculated, according to [11], for each individual in both models:

$$\begin{array}{l} u_{i}=\sum_{j=1}^{N}[I\left(w_{ij}=1\right)\left(2q_{j}\alpha_{j}\right)+I\left(w_{ij}=0\right)\left(q_{j}\alpha_{j}-p_{j}\alpha_{j}\right)\\ \;\;\;\;\;\;\;\;\;+I\left(w_{ij}=-1\right)\left(-2p_{j}\alpha_{j}\right)] \end{array}$$

where w_ij _is an indicator function of the genotype of the *jth *marker of the *ith *individual that takes the values 1, 0, -1 when the genotypes are AA, Aa or aa, respectively. Moreover, p_j _and q_j _are the allelic frequencies (A or a) for the jth marker in the training population and α is the average effect of substitution for the jth marker calculated as *α*_*j *_= *α*_*j *_under model a) and *α*_*j *_= *α*_*j *_+ *d*_*j*_(*q*_*j *_- *p*_*j*_) under model b).

### Prediction of genotype effects of future matings

The prediction of performance of future mating (G_ij_) between the *ith *and *jth *individual is performed by:

$$G_{ij}=\sum_{k=1}^{N}\left[pr_{ijk}\left(AA\right)g_{j}+pr_{ijk}\left(Aa\right)d_{j}-pr_{ijk}\left(aa\right)g_{j}\right]$$

where *pr*_*ijk *_(*AA*), *pr*_*ijk *_(*Aα*) and *pr*_*ijk *_(*aa*) are the probabilities of the genotypes AA, Aa and aa for the combination of the *ith *and *jth *individual and the *kth *marker.

### Selection strategies

Generation 1004 was formed from 25 sires and 250 dams selected from generation 1003. Two strategies of selection, carried out during five generations, were compared. In the first strategy, 25 males and 250 females were selected from 500 males and 500 females based on the prediction of breeding values from the estimation of markers effect under model a) and b), denoted and GSA and GSD, respectively. Afterwards they were mated randomly (10 dams per sire) and four sibs were obtained from each mating; the true genotypic values of the offspring were calculated.

In the second (GSD + MA), from the 6250 (25 × 250) possible matings, we chose the best 250 based on the prediction of the mating (G_ij_), and we generated four new individuals for each mating mate. The true genotypic values of the offspring were also calculated. The algorithm of searching used was the simulated annealing.

Finally, phenotypic selection was also carried out as a control, and we replicated the selection strategies by considering the true QTL as markers and the simulated effects of the additive and dominance effects of the QTL as known.

Fifty replicates of each method and strategy were performed.

## Results and discussion

### Linkage disequilibrium

**Figure 1:**
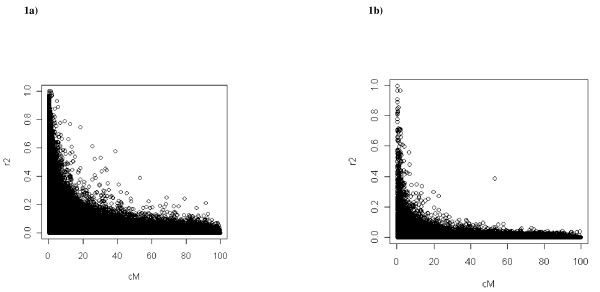
**Linkage disequilibrium for all QTL and SNPs (1a) and among QTL and SNPs (1b) as a function of the map distance**.

In generation 1003, around 8000 SNP markers and 65 QTL were segregating. The average linkage disequilibrium between adjacent polymorphisms was 0.1097. In addition, the linkage disequilibrium among the polymorphic loci (QTL and SNP) in generation 1003 measured as the square of the correlation (r^2^) is represented in Figure 1a as a function of the map distance. Besides, we have also represented in Figure 1b the r^2 ^values between QTL and SNP. Furthermore, the observed distribution of the number of SNP with different degrees of linkage disequilibrium with its nearest QTL is presented in Table 1. Thus, in generation 1003, an average of 1.39 SNP has an r^2 ^greater than 0.5 with its nearest QTL. This fact indicates that there was enough LD with QTL for selection purposes based on SNP information. Finally, the r^2 ^value among the QTL themselves attains the very low value of 0.0014.

**Table 1:** Number of SNP with different degrees of linkage disequilibrium with the QTL

**r**^**2**^	0.1-0.2	0.2-0.3	0.3-0.4	0.4-0.5	0.5-0.6	0.6-0.7	0.7-0.8	>0.8
Number	13.88	4.33	2.04	0.86	0.65	0.25	0.22	0.27
SD	8.81	3.10	2.06	1.25	0.89	0.63	0.50	0.63

Average and standard deviation (SD) (generation 1003, one replicate)

### First generation response

The results of the first generation of selection are presented in Table 2 for all the studied situations: MS (mass selection), GSA (genomic selection without dominance), GSD (genomic selection with dominance), and GSD + MA (genomic selection with dominance and mate allocation). Apart from the clear superiority of genomic selection over mass selection (MS), introduction of dominance effects in the model of evaluation (GSD) results in a clear advantage over genomic selection with an additive model (GSA). The advantage ranges from 9 to 14% of the expected response (i.e. 0.527 vs. 0.471 for h^2 ^= 0.20 and d^2 ^= 0.05). These results of the expected response are confirmed with the results of the accuracy of breeding value prediction that are also presented in Table 2. In addition, the use of mate allocation (GSD + MA) provides an additional response ranging from 6% (h^2 ^= 0.40, d^2 ^= 0.05) to 22% (h^2 ^= 0.20, d^2 ^= 0.10). In general, the superiority of GSD + MA increases as the ratio of dominance variance increases and as the heritability decreases. Both advantages are similar to those reported when dominance is included in the classical polygenic model [1,2].

**Table 2:** Comparison of selection response in the first generation with different methods

**h**^**2**^	**d**^**2**^	MS	GSA	GSD	GSD + MA	Accuracy GSD	Accuracy GSA
0.20	0.05	0.282 (0.066)	0.431 (0.042)	0.471 (0.054)	0.527 (0.048)	0.752 (0.029)	0.699 (0.036)
0.20	0.10	0.267 (0.045)	0.412 (0.059)	0.470 (0.045)	0.575 (0.060)	0.728 (0.039)	0.649 (0.062)
0.40	0.05	0.562 (0.056)	0.750 (0.052)	0.771 (0.062)	0.815 (0.058)	0.852(0.019)	0.836 (0.025)
0.40	0.10	0.557 (0.050)	0.733 (0.062)	0.754 (0.052)	0.875 (0.066)	0.850 (0.019)	0.825 (0.029)

Mass selection (MS); Genomic selection without dominance (GSA), with dominance (GSD) and genomic selection with dominance and mate allocation (GSD + MA) and the accuracy of prediction of breeding values with GSA and GSD

Furthermore, it must be mentioned that the use of a model including dominance does not give worse results even when the true simulated model is purely additive. For just one generation, the selection responses with and without dominance in the evaluation model were 0.4724 vs. 0.4670 (h^2 ^= 0.20) and 0.7832 vs. 0.7728 (h^2 ^= 0.40), respectively.

### Subsequent generation response

**Figure 2:**
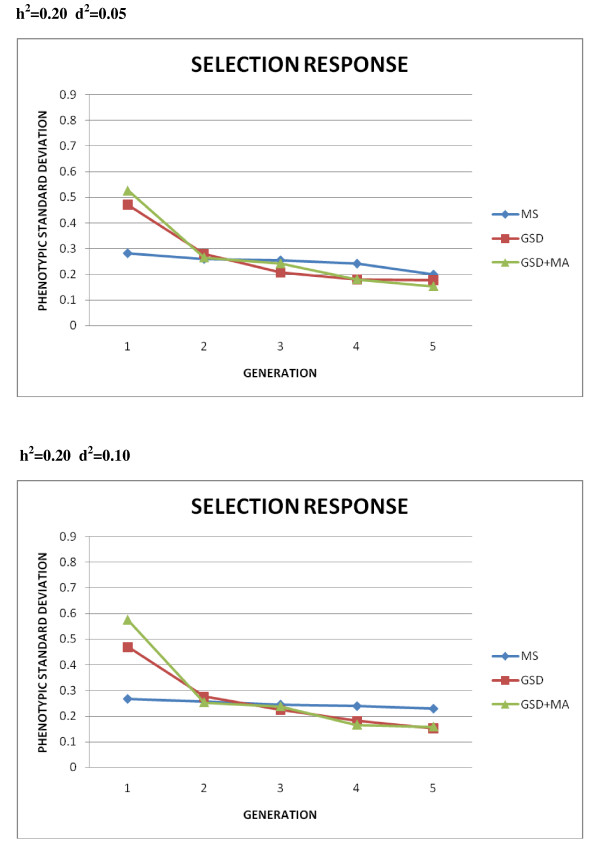
**Comparison of selection response in the first five generations for h**^**2**^** = 0.20**. Mass selection (MS); Genomic selection (GSD); Genomic selection and optimal mate allocation (GSD + MA), measured in phenotypic standard deviations

**Figure 3:**
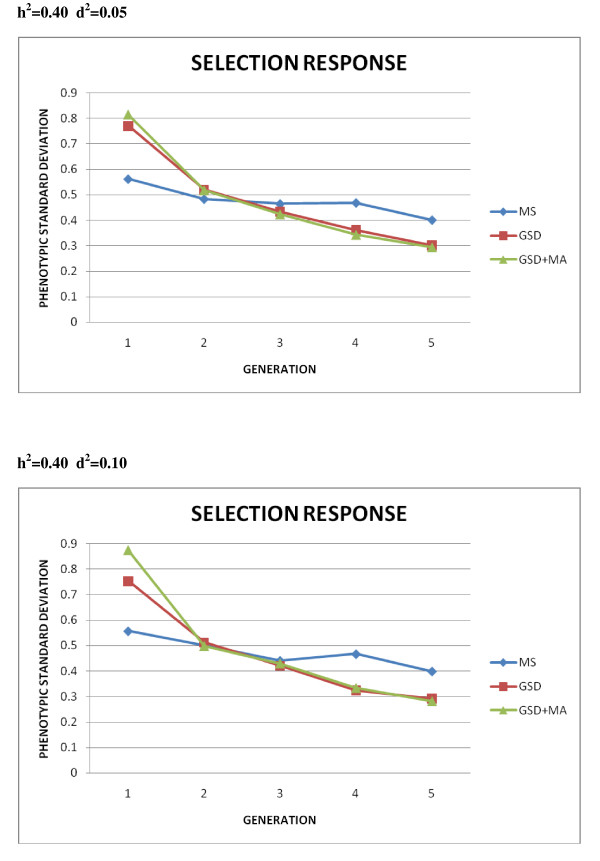
**Comparison of selection response in the first five generations for h**^**2**^** = 0.40**. Mass selection (MS); Genomic selection (GSD); Genomic selection and optimal mate allocation (GSD + MA), measured in phenotypic standard deviations

Unfortunately, the results in subsequent generations are rather discouraging for both genomic selection and mating allocation procedures. Medium term genetic responses to selection for each case of simulation are presented in Figures 2 and 3. As observed, the advantage of GSD and GSD + MA over MS presented in the previous table disappears in subsequent generations although it must be noted that MS would require extra-cost and time to record the phenotypes of candidates to selection at each generation.

**Figure 4:**
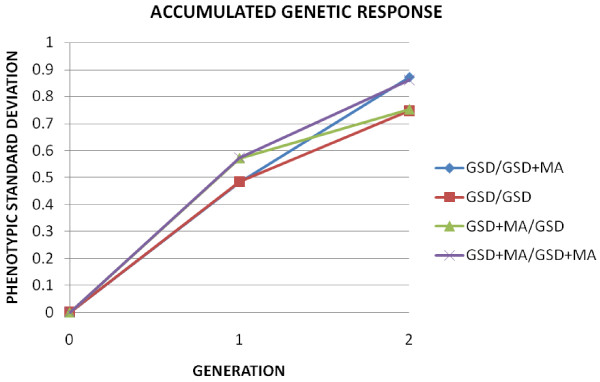
**Two generation of accumulated response to genomic selection**. Genomic selection (GSD) and Genomic selection and optimal mate allocation (GSD + MA). Mating allocation applied in one of the generations, in both or in any of them

In addition, it is notable that the increase of response due to GSD + MA over GSD is observed only in the first generation, the responses being similar from generation two to five. Thus, the advantage in terms of selection response obtained in the first generation is only maintained in the subsequent ones. However, a single generation of random mating eliminates this superiority, as shown in Figure 4, where two generations of accumulated response of the selected population are shown for four alternative selection strategies: a) GSD (1^st ^generation) - GSD (2^nd ^generation), b) GSD (1^st^) - GSD+MA (2^nd^), c) GSD + MA (1^st^) - GSD (2^nd^) and d) GSD + MA (1^st^) - GSD + MA (2^nd^).

The loss of efficiency of GS after the first generation can be attributed to the reduction of genetic variance caused by the reduced population size of the selected population and by the increase of linkage disequilibrium among the QTL as a consequence of selection, the so-called Bulmer effect [13]. In fact, the LD among QTL increases from an r^2 ^value of 0.0014 in generation 1003 to a value of 0.0032 in generation 1004.

Furthermore, additional reduction of the expected response is explained by the loss of linkage disequilibrium between the SNP and the QTL due to recombination.

### Response after random mating

In order to gain some insight in this loss of efficiency observed in Figures 2 and 3, we studied the response when GSD and GSD + MA are carried out after 0, 1, 2 and 3 previous generations with random mating and no selection in order to evaluate the consequences of reduction of linkage disequilibrium between SNP and QTL in a no selection scenario. The results are presented in Table 3. The observed selection response is eroded, but at much lower degree than in the cases where selection was carried out in previous generations.

**Table 3:** Selection response after several generations without selection (GS)

	**h**^**2**^** = 0.20 d**^**2**^** = 0.05**		**h**^**2**^** = 0.20 d**^**2**^** = 0.10**		**h**^**2**^** = 0.40 d**^**2**^** = 0.05**		**h**^**2**^** = 0.40 d**^**2**^** = 0.10**	
**GS**	**GSD**	**GSD + MA**	**GSD**	**GSD + MA**	**GSD**	**GSD + MA**	**GSD**	**GSD + MA**

1	0.432 (0.066)	0.497 (0.063)	0.415 (0.060)	0.510 (0.071)	0.711 (0.074)	0.759 (0.632)	0.716 (0.068)	0.835 (0.068)
2	0.412 (0.087)	0.478 (0.076)	0.384 (0.068)	0.465 (0.076)	0.642 (0.101)	0.708 (0.085)	0.680 (0.078)	0.753 (0.105)
3	0.398 (0.063)	0.446 (0.095)	0.361 (0.093)	0.454 (0.077)	0.614 (0.102)	0.675 (0.114)	0.630 (0.085)	0.709 (0.088)
4	0.374 (0.084)	0.420 (0.105)	0.351 (0.088)	0.438 (0.084)	0.602 (0.093)	0.640 (0.104)	0.586 (0.099)	0.701 (0.089)

Genomic selection with dominance (GSD) and genomic selection with dominance and mate allocation (GSD + MA)

**Figure 5:**
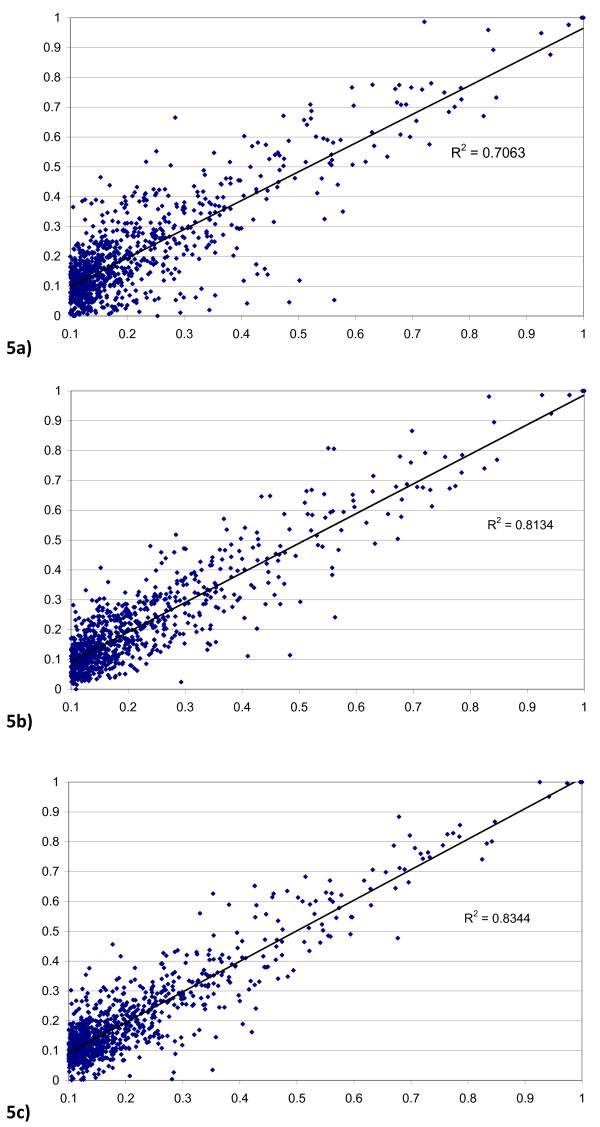
**Relationship between measures of linkage disequilibrium (r**^**2**^**) between SNP and QTL in generations 1003 and 1004 when r**^**2 **^**in generation 1003 is over 0.10 with genomic selection (5a), mas selection (5b) and without selection (5c)**.

To illustrate this fact, we calculated the linkage disequilibrium between QTL and SNP markers in generation 1003 and in generation 1004 with and without selection. Figure 5a represents the relationship between the correlation (r^2^) in generations 1003 and 1004, between every pair of QTL and SNP with r^2 ^>0.10 in generation 1003 when selection was carried out. On the contrary, Figure 5b and 5c show the same relationship in cases where individual selection or no selection occurs between generations 1003 and 1004, respectively. The LD between QTL and SNP is more conserved when selection is not carried out and when selection is performed using only phenotypic records irrespective of the distance (results not shown). Thus, the efficiency of selection by SNP markers is reduced when a previous step of genomic selection is performed.

### Known QTL genotypes and effects

In addition, we compared the results of GSD in two other different scenarios. First, we assumed that the QTL genotypes were known and we used them as markers in a Bayes A algorithm (Scenario A) and, second, we assumed the true effects of the QTL known and used them (Scenario B), the latter representing the maximum achievable response. Results are presented in Table 4. As in the previous simulations, the advantage of GSD + MA over GSD is only observed in the first generation, independently of the information used for mating prediction.

**Table 4:** Selection response after several generations of genomic selection

	**h**^**2**^** = 0.20 d**^**2**^** = 0.05**						**h**^**2**^** = 0.20 d**^**2**^** = 0.10**					
	
	GSD			GSD + MA			GSD			GSD + MA		
*Gen.*	*Markers*	*QTL*	*True*	*Markers*	*QTL*	*True*	*Markers*	*QTL*	*True*	*Markers*	*QTL*	*True*

**1**	**0.471 (0.054)**	**0.489 (0.119)**	**0.639 (0.030)**	**0.527 (0.048)**	**0.631 (0.146)**	**0.796 (0.035)**	**0.470 (0.045)**	**0.499 (0.079)**	**0.637 (0.031)**	**0.575 (0.060)**	**0.724 (0.118)**	**0.876 (0.040**
**2**	**0.280 (0.060)**	**0.363 (0.093)**	**0.492 (0.055)**	**0.265 (0.060)**	**0.348 (0.092)**	**0.493 (0.052)**	**0.275 (0.068)**	**0.343 (0.096)**	**0.489 (0.051)**	**0.253 (0.082)**	**0.317 (0.860)**	**0.467 (0.054**
**3**	**0.206 (0.061)**	**0.307 (0.085)**	**0.479 (0.058)**	**0.242 (0.054)**	**0.316 (0.096)**	**0.493 (0.058)**	**0.224 (0.082)**	**0.272 (0.105)**	**0.452 (0.061)**	**0.238 (0.060)**	**0.305 (0.097)**	**0.468 (0.059**
**4**	**0.180 (0.062)**	**0.236 (0.129)**	**0.445 (0.073)**	**0.180 (0.051)**	**0.230 (0.108)**	**0.439 (0.068)**	**0.181 (0.070)**	**0.199 (0.146)**	**0.412 (0.074)**	**0.166 (0.066)**	**0.204 (0.115)**	**0.405 (0.070**
**5**	**0.177 (0.046)**	**0.189 (0.137)**	**0.425 (0.085)**	**0.153 (0.059)**	**0.200 (0.097)**	**0.415 (0.079)**	**0.152 (0.057)**	**0.127 (0.135)**	**0.374 (0.088)**	**0.158 (0.059)**	**0.180 (0.087)**	**0.368 (0.081**

	**h**^**2**^** = 0.40 d**^**2**^** = 0.05**						**h**^**2**^** = 0.40 d**^**2**^** = 0.10**					
	
	**GSD**			**GSD + MA**			**GSD**			**GSD + MA**		

*Gen.*	*Markers*	*QTL*	*True*	*Markers*	*QTL*	*True*	*Markers*	*QTL*	*True*	*Markers*	*QTL*	*True*

**1**	**0.771 (0.062)**	**0.840 (0.063)**	**0.897 (0.048)**	**0.815 (0.058)**	**1.005 (0.056)**	**1.048 (0.046)**	**0.754 (0.052)**	**0.840 (0.065)**	**0.901 (0053)**	**0.875 (0.066)**	**1.076 (0.065)**	**1.117 (0.060)**
**2**	**0.520 (0.063)**	**0.643 (0.089)**	**0.732 (0.069)**	**0.517 (0.070)**	**0.644 (0.091)**	**0.722 (0.084)**	**0.513 (0.082)**	**0.616 (0.105)**	**0.686 (0.078)**	**0.499 (0.089)**	**0.603 (0.079)**	**0.681 (0.091)**
**3**	**0.434 (0.078)**	**0.580 (0.123)**	**0.676 (0.084)**	**0.422 (0.070)**	**0.587 (0.125)**	**0.709 (0.093)**	**0.420 (0.071)**	**0.546 (0.107)**	**0.650 (0.100)**	**0.430 (0.079)**	**0.600 (0.114)**	**0.683 (0.096)**
**4**	**0.361 (0.089)**	**0.512 (0.119)**	**0.643 (0.120)**	**0.342 (0.092)**	**0.499 (0.136)**	**0.651 (0.127)**	**0.324 (0.087)**	**0.486 (0.144)**	**0.609 (0.109)**	**0.334 (0.087)**	**0.470 (0.134)**	**0.589 (0.110)**
**5**	**0.301 (0.103)**	**0.430 (0.136)**	**0.607 (0.143)**	**0.293 (0.089)**	**0.426 (0.126)**	**0.611 (0.142)**	**0.291 (0.103)**	**0.473 (0.143)**	**0.551 (0.129)**	**0.282 (0.089)**	**0.451 (0.128)**	**0.549 (0.129)**

GSD (genomic selection with dominance) and GSD + MA (genomic selection with dominance and mate allocation) and using SNP markers (*markers*), QTL genotypes as markers (*QTL*), and known QTL effects (*True*)

If we examine the increase of response due to MA in the first generation, in Scenario A (QTL genotypes known) it ranges from 19% (h^2 ^= 0.40 and d^2 ^= 0.05) to 45% (h^2 ^= 0.20 and d^2 ^= 0.10) and in Scenario B (QTL genotypes and effects known) from 17% (h^2 ^= 0.40 and d^2 ^= 0.05) to 38% (h^2 ^= 0.20 and d^2 ^= 0.10). Although the percentage of increase over GSD is greater in Scenario A, the absolute value of extra response due to MA is bigger in Scenario B, as expected when maximum information is available. Success of MA is due to the possibility of predicting the genotype of future offspring and of estimating the additive and dominance effects. The first challenge is accomplished even in Scenario A, which shows a higher relative superiority than Scenario B. In addition, these extra genetic responses are greater than the ones shown in Table 2, when SNP genotypes are used to predict additive and dominance effects.

Furthermore, a strong reduction in the genetic response is observed between the first and the second generations for every scenario. However, the response is maintained at a higher degree when QTL effects are known than when SNP or QTL effects are estimated. As expected, the scenario in which QTL genotypes are known but their effects need to be estimated, provides an intermediate response.

## Conclusions

Introduction of dominance effects in genetic evaluation is easier to achieve in the whole-genome evaluation scenario than in the classical polygenic model, where potential parental combinations have to be defined and evaluated. Introduction of dominance effects in models of whole-genome evaluation provides two main results. First, it increases the accuracy of prediction of breeding values and second, it makes it possible to obtain an extra response by the appropriate design of future matings using mate allocation techniques.

Thus, mate allocation is recommended in the genetic management of populations under selection by whole-genome evaluation procedures, although the potential extra response is achieved only in the first generation and then maintained afterwards.

Our results also show that in most scenarios of genomic selection a continued collection of phenotypic data and re-evaluation of the additive and dominance effects of markers will be required, because the ability of predicting breeding values is greatly reduced when selection is carried out.

## Competing interests

The authors declare that they have no competing interests.

## Authors' contributions

LV wrote the main computer programs and ran them. Both authors wrote and approved the final manuscript.
